# Supplementary material for: MetaQTL: a package of new computational methods for the meta-analysis of QTL mapping experiments
Source: BMC Bioinformatics. 2007 Feb 8;8:49. doi: 10.1186/1471-2105-8-49 (PMC1808479; doi:10.1186/1471-2105-8-49)
Supplement: Additional File 7 — MetaQTL Package : jar file and tutorial. This Zip archive contains both the MetaQTL JAR file and the files of the tutorial. [file 1471-2105-8-49-S7.zip › org.inra.metaqtl/doc/org/metaqtl/main/package-summary.html]

org.metaqtl.main


|  |  |  |
| --- | --- | --- |
| **Overview** \| **Package** \| Class \| **Use** \| **Tree** \| **Deprecated** \| **Index** \| **Help** | |  |
| **PREV PACKAGE**   **NEXT PACKAGE** | **FRAMES**    **NO FRAMES**     **All Classes** |  |


---

## Package org.metaqtl.main

| **Class Summary** | |
| --- | --- |
| **A2Xml** |  |
| **CmdLineParser** | Largely GNU-compatible command-line options parser. |
| **CmdLineParser.Option** | Representation of a command-line option |
| **CmdLineParser.Option.BooleanOption** |  |
| **CmdLineParser.Option.DoubleOption** | An option that expects a floating-point value |
| **CmdLineParser.Option.IntegerOption** | An option that expects an integer value |
| **CmdLineParser.Option.LongOption** | An option that expects a long integer value |
| **CmdLineParser.Option.StringOption** | An option that expects a string value |
| **ConsMap** |  |
| **InfoMap** |  |
| **MapView** |  |
| **MetaDB** |  |
| **MetaMain** |  |
| **MMapView** |  |
| **MQTLView** |  |
| **QTLClust** |  |
| **QTLClustInfo** |  |
| **QTLModel** |  |
| **QTLProj** |  |
| **QTLTree** |  |
| **Xml2A** |  |

| **Exception Summary** | |
| --- | --- |
| **CmdLineParser.IllegalOptionValueException** | Thrown when an illegal or missing value is given by the user for an option that takes a value. |
| **CmdLineParser.NotFlagException** | Thrown when the parsed commandline contains multiple concatenated short options, such as -abcd, where one or more requires a value. |
| **CmdLineParser.OptionException** | Base class for exceptions that may be thrown when options are parsed |
| **CmdLineParser.UnknownOptionException** | Thrown when the parsed command-line contains an option that is not recognised. |
| **CmdLineParser.UnknownSuboptionException** | Thrown when the parsed commandline contains multiple concatenated short options, such as -abcd, where one is unknown. |

---


|  |  |  |
| --- | --- | --- |
| **Overview** \| **Package** \| Class \| **Use** \| **Tree** \| **Deprecated** \| **Index** \| **Help** | |  |
| **PREV PACKAGE**   **NEXT PACKAGE** | **FRAMES**    **NO FRAMES**     **All Classes** |  |


---
